# The effects of fatigue and oxidation on contractile function of intact muscle fibers and myofibrils isolated from the mouse diaphragm

**DOI:** 10.1038/s41598-019-39353-5

**Published:** 2019-03-14

**Authors:** M. Angela Bagni, Barbara Colombini, Marta Nocella, Claudio Pregno, Anabelle S. Cornachione, Dilson E. Rassier

**Affiliations:** 1 Dept Experimental and Clinical Medicine, University of Firenze, Florence, Italy; 2 Dept Kinesiology and Physical Education, Faculty of Education, McGill University, Montreal, Canada

## Abstract

The goal of this study was to investigate the effects of repetitive stimulation and the oxidant H_2_O_2_ on fatigue of diaphragm intact fibers and in myofibrils measured with different Ca^2+^ concentrations. Intact fibers were isolated from mice diaphragm, and twitch and tetanic contractions (500 ms duration) were performed at different frequencies of stimulation ranging from 15 Hz to 150 Hz to establish a force-frequency relation before and after a fatigue and recovery protocol, without or after a treatment with H_2_O_2_. Fatigue was induced with isometric contractions (500 ms, 40 Hz) evoked every 0.8 seconds, with a total of 625 tetani. After the fatigue, the force recovery was followed by invoking tetanic contractions (500 ms, 40 Hz) every 1 min, with a total duration of 30 min. Individual myofibrils were also isolated from the mouse diaphragm and were tested for isometric contractions before and after treatment with H_2_O_2_ and NAC. In a second series of experiments, myofibrils were activated at different pCa (pCa = −log_10_ [Ca^2+^]), before and after H_2_O_2_ treatment. After 15 minutes of H_2_O_2_ treatment, the myofibrillar force was decreased to 54 ± 12% of its control, maximal value, and a result that was reversed by NAC treatment. The force was also decreased after myofibrils were treated with H_2_O_2_ and activated in pCa ranging between 4.5 and 5.7. These results suggest that fatigue in diaphragm intact fibers and at the myofibrils level is caused partially by oxidation of the contractile proteins that may be responsible for changing the force in various levels of Ca^2+^ activation.

## Introduction

During a period of prolonged activity, the diaphragm muscle develops fatigue and the force output declines^1,2^. Fatigue in diaphragm is important, as it underlies a decrease in physiological function, and it can lead to the development of hypercapnic respiratory failure^1,3,4^. Prolonged fatigue is also present after exhaustive respiratory work in the diaphragm of healthy individuals, which can cause respiratory failure^5^.

The mechanism behind diaphragm fatigue remains under investigation. Recent studies suggest that oxygen-derived free radicals are produced in the respiratory muscles in response to strenuous and repeated contractions. This production of reactive oxygen intermediates leads to oxidative stress of muscle proteins, and is directly associated with fatigue of limb^6,7^ and ventilatory^8,9^ muscles. There is an increase in the production of free radicals in respiratory muscles after electrically induced contractions^10,11^ and after inspiratory loading of whole animals^8,12,13^. In addition, several studies have shown that it is possible to attenuate the rate of development of diaphragmatic fatigue during electrically-induced contractions by administration of free radical scavengers, which prevent free radical-mediated muscle dysfunction^9^.

The difficulty in defining a precise mechanism for diaphragm fatigue is partly because studies have been performed in a diversity of systems, ranging from permeabilized fibers, intact muscles, healthy humans and patients suffering diseases such as cardiomyopathy. These models are important to investigate muscle fatigue, but do not allow for precise measurements of the specific, cellular force. Furthermore, the environment surrounding the fibers during experiments is difficult to control. In this study, we took advantage of two preparations that we developed in our laboratories that allow the evaluation of the cellular and sub-cellular mechanisms of fatigue: intact living fibers and isolated myofibrils. Intact fibers permit the evaluation of diaphragm mechanisms using the smallest contractile system that still maintains the main physiological characteristics^14–18^. The fibers can be activated by electrical stimulation, which initiates action potentials, and can be experimented for many contractions, which allows a proper investigation of fatigue^19,20^. Myofibrils allow the control of the media surrounding the preparations while rapidly activating and relaxing the contractile system, and a precise measurement of specific force given its small cross-sectional area (~1 μm)^21–24^. Using these preparations, we defined the contractile characteristics of diaphragm and looked into the effects of an oxidant in the development of force and fatigue, and in the myofibrillar response to different levels of Ca^2+^ activation.

## Methods

In this study, we used small bundles with intact diaphragm fibers and isolated diaphragm myofibrils from the mouse. The animal protocol for the work with mice fibers was undertaken in compliance with the guidelines of the European Communities Council Directive 2010/63/UE and the recommendations for the care and use of laboratory animals and it was approved by the animal care Committee of the University of Florence (Italy) and Italian health Ministry (Authorization 708/2017-PR). The animal protocol for the mice myofibrils was approved by the Animal Care Committee at McGill University and the Canadian Council on Animal Care (Reference number: 20122–23).

### Intact fiber preparation

Experiments with intact fibers were performed using CD1 mice (Envigo, RMS Srl, Udine, Italy). Animals were housed with controlled temperature (21–24 °C) and a 12–12 h light-dark cycle. Food and water were provided ad libitum. Mice (7–8 months old) were killed by prompt cervical dislocation to minimize animal suffering. The diaphragm muscle was quickly excised and placed in Tyrode solution of the following composition (mM): NaCl, 121; KCl, 5; CaCl_2_, 1.8; MgCl_2_, 0.5; NaH_2_PO_4_, 0.4; NaHCO_3_, 24; glucose, 5.5; EDTA, 0.1 and bubbled with 5% CO_2_–95% O_2_ which gave a pH of 7.4. Fetal calf serum (0.2%) was routinely added to the solution.

Small bundles of up to ten intact fibers were dissected from the diaphragm as described previously^14^. The use of small bundles of fibers (called fibers in this paper, for simplicity) offers ideal conditions for mechanical measurements. The dissection was performed manually under a stereomicroscope with a fine pair of scissors and needles taking care to avoid stretching and to obtain preparations clean of debris from dead fibers. Small aluminum T-shaped clips were fixed to tendons as close as possible to the fiber ends. The fibers were transferred to a temperature controlled experimental chamber (801C/1900, Aurora Scientific, Toronto CA) and mounted on an inverted microscope (Axiovert 40CFL, Zeiss DE). The clips attached the fibers horizontally between the lever arms of a capacitance force transducer (405A, Aurora Scientific) and a length controller (322 Aurora Scientific). Fibers were superfused continuously with oxygenated Tyrode solution by means of a peristaltic pump.

The experiments were performed at 27–28 °C in Tyrode solution. After a 15 min thermo-equilibration period in the bath, bipolar stimuli (1 ms duration and 1.5 times threshold strength) were applied across the fibers by means of two platinum-plate electrodes mounted parallel to the bundle via a high power bipolar stimulator (701C Aurora Scientific). After a test of viability, the desired sarcomere length was set by adjusting the fiber length. Tetanic stimulation (500 ms duration) was applied at 1 min intervals using the minimum frequency necessary to obtain a fused contraction (75–125 Hz). The fibers were set at a length at which tetanic force was maximal, which in the case of the current experiments corresponded to a mean sarcomere length of approximately 2.70 µm. Tetani were given for a period of equilibration of approximately 10 min, if the maximum tetanic force (P_0_) decreased by >15%, the fibers were discarded.

The resting fiber length, the bundle largest and smallest diameters, and the resting sarcomere length were measured using the microscope fitted with 20× eyepieces and a 5× or 40× dry objective in the experimental chamber. The preparation length (l_0,_ clip to clip) was 11.19 ± 0.37 mm (mean ± S.E.M, n = 19), of which 9.90 ± 0.34 mm composed the fiber length and the remaining (1.29 ± 0.14 mm) the tendons attachments. The cross-sectional area of the bundles was calculated as a*b*π/4 where a and b are the average values of the width and the vertical height of the preparations respectively, measured at 2–3 different points along the bundles.

**Figure 1:**
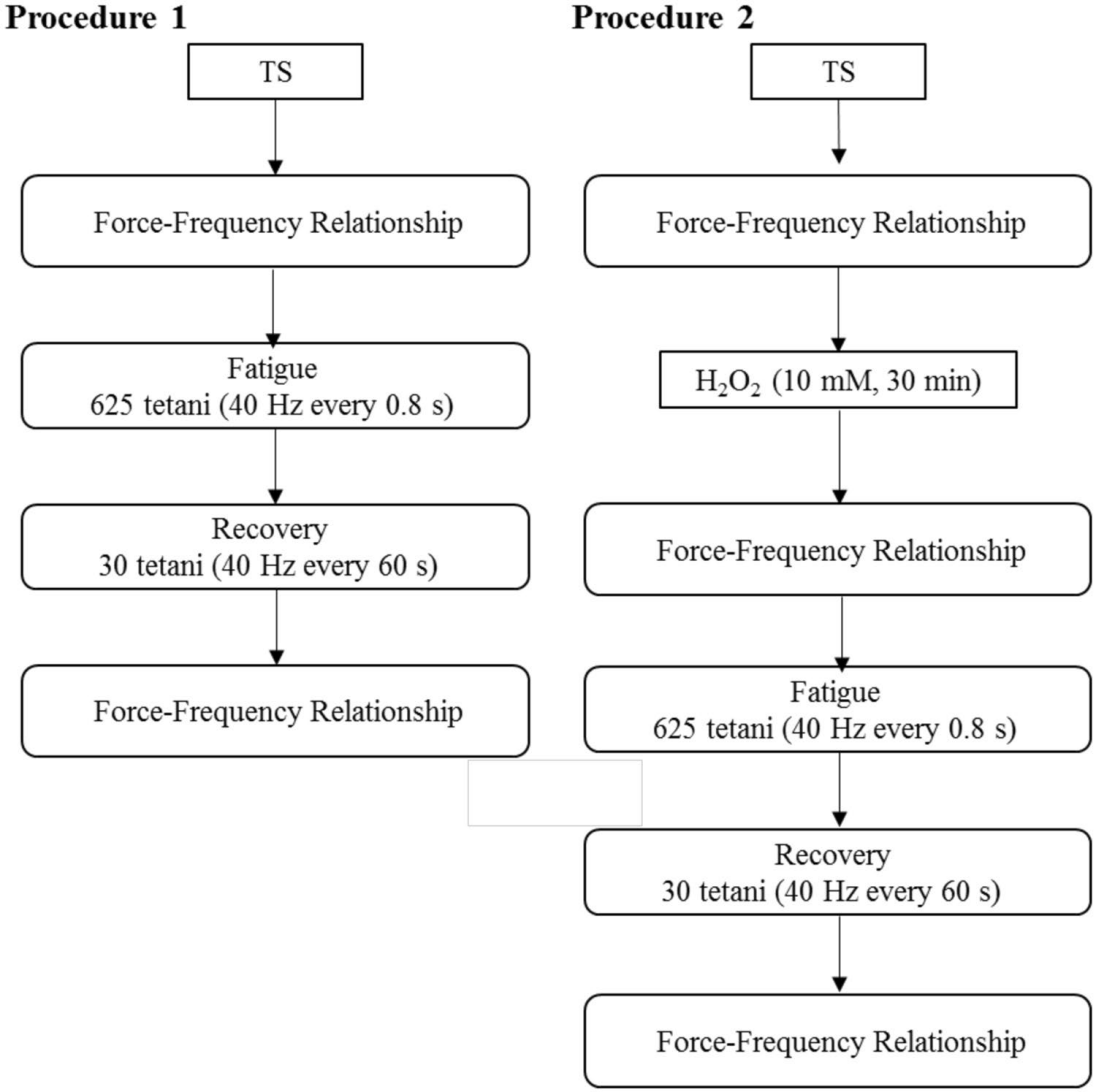
Flow chart depicting the two sets of experiments conducted with single intact fibers isolated form the diaphragm. TS = Tyrode solution.

Twitch (1 stimulus, 1 ms duration) and tetanic contractions (500 ms duration, 15 Hz, 30 Hz, 40 Hz, 50 Hz, 75 Hz, 100 Hz, 125 Hz and 150 Hz) were evoked with 1 min rest intervals. Such procedure was used to establish a force-frequency relation for the fibers investigated in this study. Following, one of the two procedures were used: (i) a fatigue protocol was delivered, or (ii) fibers were treated with H_2_O_2_ (10 µM) for 30 min. In the presence of H_2_O_2_, a force-frequency relation was established, followed by the fatigue protocol. Fatigue was always induced using isometric contractions (500 ms, 40 Hz) evoked every 0.8 seconds and the protocol was stopped after 625 tetani. After the fatigue protocol, the force recovery was followed by invoking tetanic contractions (500 ms, 40 Hz) every 1 min, with a total duration of 30 min. After the fatigue and recovery protocols, the force-frequency relation was established again. The protocols are summarized in the flow chart shown in Fig. 1. Both protocols induced a large number of contractions, but it is known that the maximal force developed by intact muscle fibers remains stable for many hours when the fibers are stimulated at regular intervals (1–2 minutes) with tetanic stimulation frequency (40–150 Hz)^25,26^. The output of the force transducer and the length controller were acquired in real time using an integrated PC board (NI6221, National Instruments USA). The experimental data were displayed and successively analyzed with a dedicated controlled software (600A Digital controller, Aurora Scientific, CA).

#### Data analysis

The forces produced by the fibers under the different conditions used in this study (control, treatment with H_2_O_2_) at different frequencies of stimulation were analyzed by a two-way analysis of variance (ANOVA) for repeated measures. The forces produced by the fibers under the different conditions at different times during the fatigue protocol were also analyzed by a two-way ANOVA for repeated measures. When ANOVA revealed a statistical difference for the interaction between factors or for main factor effects, post-hoc comparisons were performed using Bonferroni’s test. All results in this paper are shown as mean ± S.E.M. A level of significance of p < 0.05 was used for all analyses.

### Myofibril preparation

Experiments with myofibrils were performed with CD1 mice obtained from Charles River Canada. Small muscle bundles of the diaphragm muscle were dissected, tied to wood sticks, and chemically permeabilized following standard procedures used in our laboratory^21^. Muscles were incubated in rigor solution (pH = 7.0) for approximately 4 hours, after which they were transferred to a rigor: glycerol (50:50) solution for approximately 15 hours. The samples were subsequently placed in a fresh rigor: glycerol (50:50) solution with the addition of a cocktail of protease inhibitors (Roche Diagnostics, USA) and stored in a freezer (−20 °C) for at least seven days. On the day of the experiments, small pieces of the samples were homogenized following standard procedures^22–24^, which resulted in a solution containing isolated myofibrils. The myofibrils were transferred to the experimental setup, which contained a system for detection of atomic force cantilever (AFC) displacements^27^.

#### Measurements of myofibril forces

The myofibrils were attached between the AFC and a rigid glass needle. A multichannel fluidic system connected to a double-barreled pipette was used for fast activation and deactivation of the myofibrils. During the experiments, the position of the double-barreled pipette was rapidly switched to change the solutions surrounding the myofibrils (between pCa 4.5 and pCa 9.0; pCa = −log_10_ [Ca^2+^]). Such procedure allows for a fast activation and relaxation of the myofibrils without damages to the preparation, and has been explained in details in previous studies^22,24,27^. Briefly, a laser is shined upon and reflects from the AFC, which acts as a force transducer. When an attached myofibril is shortened due to activation it causes AFC deflection, which is detected and recorded using a newly developed optical system with a high time resolution in the order of milliseconds^27^. Since the stiffness of the AFC (K) was known and we measured the amount of cantilever displacement (Δd), the force (F) could be calculated as F = K⋅Δd.

Under high magnification, the contrast between the dark bands of myosin (A-bands) and the light bands of actin (I-bands) provided a dark-light intensity pattern, representing the striation pattern produced by the sarcomeres, which allowed measurements of sarcomere length during the experiments. The sarcomere length was measured with a video camera connected to the right-side port of the microscope. Once the myofibrils were attached between the AFC and the micro-needle, they were adjusted to an average sarcomere length of 2.7 µm. Throughout the experiments, homogeneity of sarcomere length was accessed to detect potential damage in the myofibrils. When non-uniformity of sarcomere lengths formed during activation did not recover into a regular striation pattern before the next activation, the experiment was stopped and the myofibril was discarded from further analysis.

The myofibril length was more variable than the intact fiber length during the experiments, as myofibrils of distinct lengths were produced during the homogenization protocol and were selected based on their appearance; the length was 26.12 μm ± 8.71 (mean ± S.E.M, total n in all protocols used in this study = 32). The cross-sectional area of the myofibrils, calculated using a method similar to that used in intact fibers (see above) was 1.10 μm ± 0.11.

Three protocols were used in the experiments with myofibrils. (i) The myofibrils (n = 13) were activated in a pCa of 4.5 to produce a fixed-end, isometric contraction for 15–20 sec. After a short interval, the myofibrils were incubated in a solution contacting H_2_O_2_ for 30 min (10 μM). During this period, the myofibrils were activated (pCa 4.5) at 5 min, 15 min and at the end, after washing the preparation with resting solution (pCa 9.0). Finally, the myofibrils were treated with NAC (5 μM) for 30 min and activated again to produce maximal force. (ii) The myofibrils (n = 7) were activated in a pCa of 4.5 to produce a fixed-end, isometric contraction for 15–20 sec. After a short interval, the myofibrils were incubated in a solution contacting H_2_O_2_ for 30 min (10 μM). During this period, the myofibrils were activated (pCa 4.5) at 5 min, 15 min and at the end, after washing the preparation with resting solution (pCa 9.0). Finally, the myofibrils were rested for 30 min without NAC treatment, and activated again to produce maximal force. (iii) The myofibrils (n = 12) were activated in pCa varying between 4.5 and 9.0 in order to produce a force-pCa relation. Then the myofibrils were incubated for 15 min in solution contacting H_2_O_2_ for 30 min, and activated in pCa 5.7, 5.5 and 4.5 (random order).

#### Myofibril solutions

The rigor solution (pH 7.0) used to store the myofibrils was composed of (in mM): 50 Tris, 100 NaCl, 2 KCl, 2 MgCl_2_, and 10 EGTA. The relaxing solution used for muscle dissection (pH 7.0) was composed of (in mM) 100 KCl, 2 EGTA, 20 imidazole, 4 ATP, and 7 MgCl_2_. The experimental solutions used during the experiments (pH 7.0) was composed of (in mM) 20 imidazole, 14.5 creatine phosphate, 7 EGTA, 4 MgATP, 1 free Mg^2+^, free Ca^2+^ ranging from 1 nM (pCa 9.0) to 32 μM (pCa 4.5), and KCl to adjust the ionic strength to 180 mM. The final concentrations of each metal-ligand complex were calculated using a computer program based on previous studies^24,28,29^. All experiments were performed at 15 °C.

#### Data analysis

The forces produced by the myofibrils in the different conditions in protocols (i) and (ii) were analyzed by a one-way ANOVA for repeated measures. The force developed in pCa 5.7, 5.5 and 4.5 were compared between the two groups (control and after H_2_O_2_ treatment) in protocol (iii) using a one-way ANOVA for repeated measures. All data are shown as mean ± S.E.M. A level of significance of p < 0.05 was used for all analyses.

## Results

### Fiber mechanics

**Figure 2:**
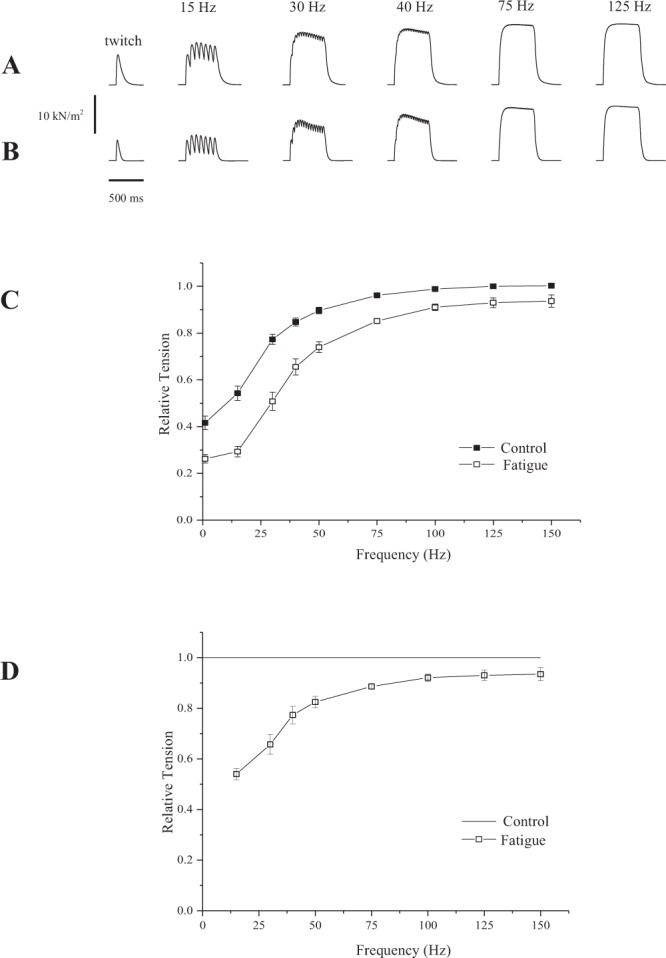
Isometric contractions recorded with single fibers from the mouse at different frequencies of stimulation, before (**A**) and after (**B**) fatigue. (**C**) Force-frequency relation before and after fatigue, with values normalized by the maximum force obtained during the protocol at 125 Hz. (**D**) Force-frequency relation with values normalized for the maximum force obtained in each frequency of stimulation. TS = Tyrode solution. Values are mean ± S.E.M.

Figure 2A shows traces of force produced by intact fibers during different frequencies of stimulation, ranging from a twitch contraction to 125 Hz. When activated at frequencies ≥40 Hz, the fibers developed force rapidly reaching a plateau and stabilizing for as long as activation persisted (Fig. 2A), similar to what has been observed with single fibers from other skeletal muscles^18,19,25,30^. The force-frequency relation for the diaphragm fibers examined in this study (Fig. 2C) resembles that observed in previous studies with single skeletal muscle fibers, with a maximal force obtained with stimulation frequencies between 75 Hz and 125 Hz^31,32^.

Figure 2B also shows experiments in which the fibers were activated at different frequencies of stimulation after they were fatigued and allowed to recover for 30 min (i.e., following protocol described in the Methods). The fatigue followed a well-described protocol of repeated stimulation over several or 625 tetanic contractions^19,25,30^. After fatigue and recovery, the force never reached the same maximum values as before fatigue (Fig. 2B,C), even at maximum frequencies of stimulation used in this study, which caused a marked downward shift in the force frequency relation (main overall effect = p < 0.001). Figure 2D shows the force normalized by the maximum value obtained in each frequency of stimulation. The force was decreased mostly at low frequencies of stimulation.

**Figure 3:**
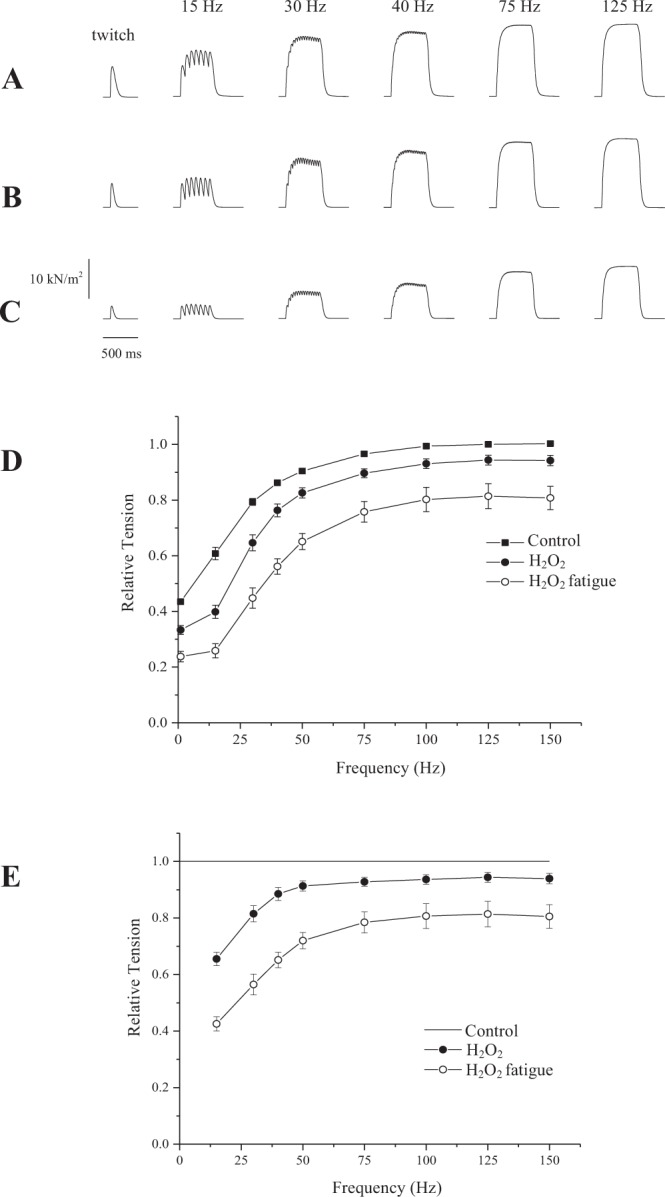
Isometric contractions recorded with single fibers from the mouse at different frequencies of stimulation, before any treatment (**A**), after treatment with H_2_O_2_ (**B**), and after fatigue in the presence of H_2_O_2_ (**C**,**D**) Force-frequency relation before and after treatment with H_2_O_2_ and fatigue, with values normalized by the maximum force obtained during the protocol at 125 Hz. (**E**) Force-frequency relation with values normalized for the maximum force obtained in each frequency of stimulation. TS = Tyrode solution. Values are mean ± S.E.M.

In other series of experiments, the fibers were treated with H_2_O_2_ before fatigue. Figure 3A shows the contraction recorded at control conditions, in the beginning of the protocol, showing results that were similar to those obtained during the first series of experiments (Fig. 2A). After treatment with H_2_O_2_, the force decreased significantly in all frequencies investigated in this study (Fig. 3B). The contractions looked similar to those elicited before treatment, but with a smaller force production. As shown in Fig. 3D there was a downward shift in the entire force-frequency relation (main overall effect = p < 0.001). Figure 3C also shows contractions obtained from the same fibers after they were treated with H_2_O_2_ and fatigued with the same protocol as used in untreated fibers. The force decreased further, reaching levels that were significantly lower than before fatigue and H_2_O_2_ treatment. The downward shift in the force-frequency curve is even more accentuated (Fig. 3D). Figure 3E shows the force normalized by the maximum value obtained in each frequency of stimulation, after treatment with H_2_O_2_ and H_2_O_2_ plus fatigue. The force was decreased mostly at low frequencies of stimulation.

**Figure 4:**
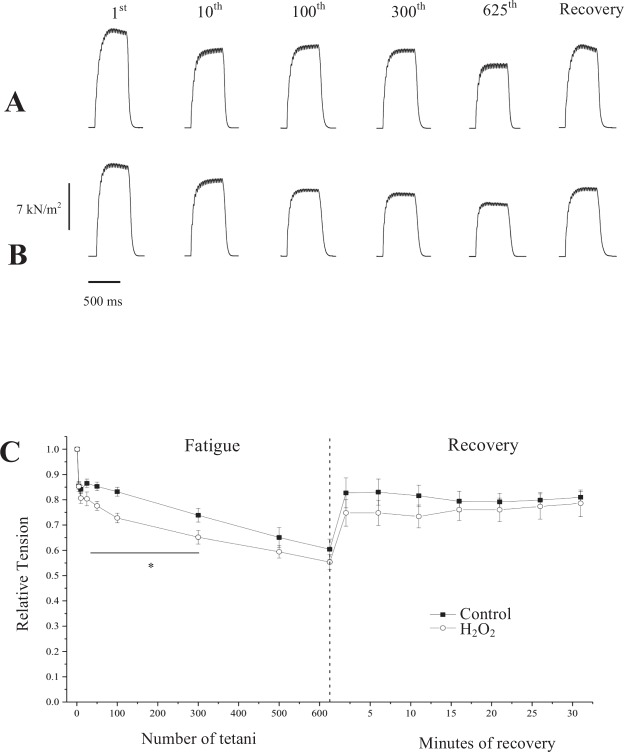
(**A**) Isometric contractions recorded during the fatigue protocol, before and after H_2_O_2_ treatment. We are showing the 1^st^ (control) 10^th^, 100^th^, 300^th^, 625^th^ contractions during the fatigue protocol and the contraction recorded after recovery. (**B**) Pattern of changes in force during the fatigue protocol and recovery. TS = Tyrode solution. Values are mean ± S.E.M.

Figure 4A shows some of the contractions that were recorded during the fatigue protocol, and after recovery in control fibers (upper traces) and after H_2_O_2_ treatment fibers (lower traces). At the end of the fatigue protocol, the force reached levels that varied between ~58% to ~65% of the force produced at the beginning of the protocol after H_2_O_2_ treatment or without treatment, respectively. The force was always smaller after treatment with H_2_O_2_ for any given time during the protocol, including the recovery (Fig. 4A). Figure 4B shows the mean values of force changes developed during the repeated contractions that led to fatigue in control fibers, or after treatment with H_2_O_2_, and also during recovery. Since the force values were different at the beginning of the protocol, we normalized all the force values relative to the maximal force developed at 40 Hz before the fatigue protocol (P_40_), for better compare the conditions (with or without H_2_O_2_ treatment). After a marked change in force observed after the first 10–15 tetani in both conditions, the force decreased substantially more in fibers treated with H_2_O_2_, but with a pattern of changes that was relatively similar across the groups. At the end of fatigue, in untreated fibers (n = 9), force decreased to 0.61 ± 0.04 P_40_ whereas after treatment with H_2_O_2_ (n = 6) the fibers were largely more fatigable from the 25nd tetanus. All fibers endured the full fatiguing stimulation protocol after H_2_O_2_ treatment, and the mean force in the last tetanus was 0.55 ± 0.03 P_40_. Analysis of variance detected that in the presence of H_2_O_2_, fatigue development was significantly increased (p < 0.05) during the first 300 tetanic contractions. After 1 min of recovery, tetanic force reached 0.83 ± 0.06 and 0.75 ± 0.05 the pre-fatigue value in control or after H_2_O_2_ treatment fibers, respectively (Fig. 4C). All the fibers reached the maximum force recovery value in a few minutes after the end of fatigue and then it remains almost constant. At the end of the recovery period, the tetanus force was not significantly different in control (0.81 ± 0.02 P_40_) or after H_2_O_2_ treatment fibers (0.79 ± 0.05 P_40_).

It is worth noting that even after recovery, the force was decreased further in all frequencies of stimulation tested in this study (Figs 2C,D and 3D,E); there was a main statistical effect observed after analysis of variance (p < 0.001). Overall, the results suggest that oxidation decreases the force in all frequencies of stimulation and that fatigue decreases the force further, independently of the stimulation frequency (Fig. 3D,E).

### Myofibril mechanics

The decrease in force observed after treatment of the fibers with H_2_O_2_ was assumedly associated with oxidation of the contractile apparatus. Therefore, we performed addition experiments in which we used isolated myofibrils from the diaphragm, preparations that allow the control of the experimental environment and the Ca^2+^ concentrations surrounding the preparations. In this way, we can measure the force response to different levels of Ca^2+^ activation.

**Figure 5:**
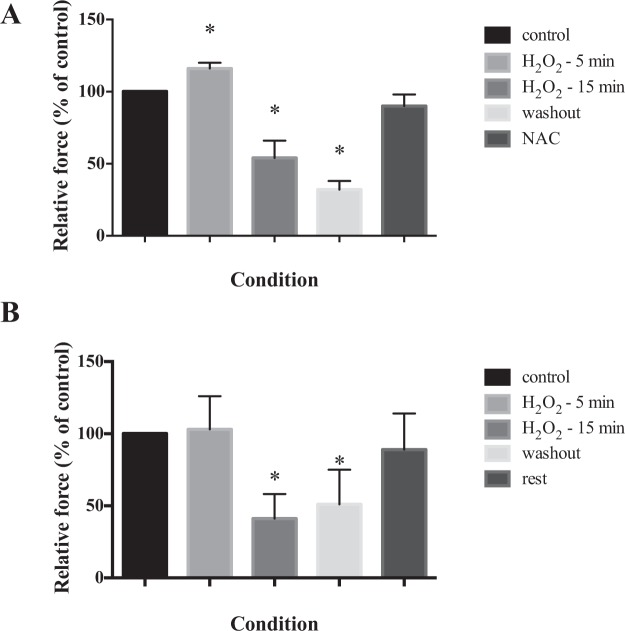
The tension produced by myofibrils in response to different Ca^2+^ concentrations, and after treatment with H_2_O_2_ and NAC. In all graphs, values are mean ± S.E.M. The asterisks (*) in panels A and B denote a significant difference from the control condition. (**A**) Isometric force produced by myofibrils (n = 13) in response to high concentrations of Ca^2+^ (pCa = 4.5) without treatment, after 5 and 15 min of H_2_O_2_ treatments, after washing out H_2_O_2_, and after treatment with NAC. (**B**) Isometric force produced by myofibrils (n = 7) in response to high concentrations of Ca^2+^ (pCa = 4.5) without treatment, after 5 and 15 min of H_2_O_2_ treatments, after washing out H_2_O_2_, and after an additional rest period of 30 minutes.

Figure 5A shows the values of force produced by contractions developed by isolated myofibrils in different solutions. The force produced by the myofibril before treatment is similar to that observed in previous studies using myofibrils from skeletal muscles at a similar temperature^21,24,33,34^. After treatment with H_2_O_2_ for 5 min, the force increased slightly (Fig. 5A), but significantly (p < 0.05). However, after 15 min of incubation in H_2_O_2_, the force decreased substantially compared to the force produced at the begging of the experiments (p < 0.01), and by the end of the incubation period it reached levels of ~30% of maximal, a result that was consistent across preparations (Fig. 5A). The result directly fits with the results of a previous another that examined the effects of H_2_O_2_ on permeabilized fibers from the rabbit diaphragm^35^, a preparation with similar characteristics as the myofibrils used in this study. After treatment with H_2_O_2_, the myofibrils were washed and then treated with the anti-oxidant N-acetylcysteine (NAC) before being activated again. NAC has been used in a variety of experiments with intact muscle bundles and whole muscle preparations^13,36,37^, and has been shown to inhibit the rate of fatigue in the diaphragm. However, it has not been tested in permeabilized fibers. In our study, the force levels returned to levels close to control levels after NAC treatment, similar to the force developed by the myofibrils at the beginning of the experiments. However, when we repeated the same experiment protocol but did not use NAC (Fig. 5B), and just left the fibers resting for 30 min after treatment with H_2_O_2_, the force also recovered to levels that were not significantly different from the control condition. This result suggests that NAC may not exert a prominent effect on permeabilized myofibrils, since its effects are mostly on the membrane of muscle fibers, a permeable source of cysteine that can be used as a substrate for the increased production of the endogenous antioxidant glutathione.

**Figure 6:**
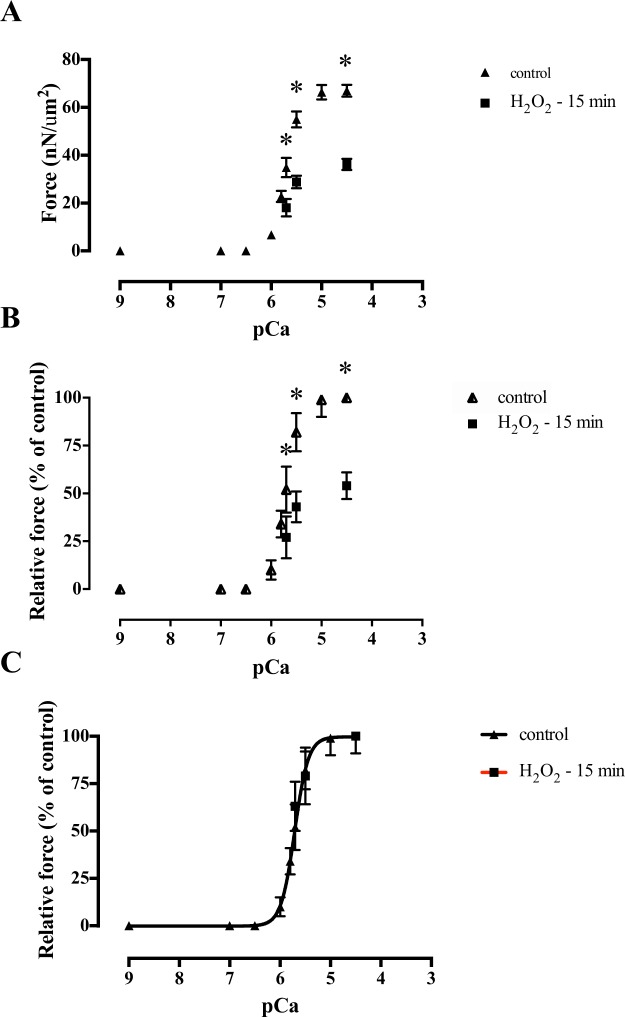
The force-pCa relation in myofibrils (n = 12) before and after treatment with H_2_O_2_ for 15 min. In all graphs, values are mean ± S.E.M. The asterisks (*) indicate a significant difference between the two conditions in a given pCa. (**A**) Force obtained by the two groups normalized by the myofibril cross-sectional area. (**B**) Force obtained in the two groups normalized by the maximal force obtained during the control experiments. (**C**) Force normalized by the maximal force obtained in each group. The data for each group was fitted with the Hill equation: Y = 1/(1 + 10^nH (pCa−pCa50)^), where pCa_50_ is the pCa where force reaches half-maximal force, and *n*H is the Hill coefficient, representing the steepness of the force-pCa relation.

In a third series of experiments, we activated the myofibrils using different Ca^2+^ concentrations to build a force-Ca^2+^ relation (Fig. 6A–C). Although we are not aware of previous experiments with isolated diaphragm myofibrils, the force-pCa relation obtained in our experiments resembles that observed in previous studies with skeletal muscles (Fig. 6A,B). We then repeated contractions in selected Ca^2+^ concentrations, but after 15 min of H_2_O_2_ treatment, and observed that the force was significantly decreased in all pCa tested (pCa 5.7, p < 0.05; pCa 5.5, p < 0.001; pCa 4.5, p < 0.001) (Fig. 6A,B). We also compared the forces developed in the different Ca^2+^ concentrations after normalizing the values by the maximal force obtained in each group, instead of normalizing the values by the maximal force produced at the control situation (Fig. 6C). The force-pCa curves from the two groups virtually overlap, suggesting that H_2_O_2_ did not change the Ca^2+^ sensitivity of the myofibrillar contractile apparatus. We fitted the data in each experiment with a Hill sigmoidal curve (Prism 6, GraphPad Software Inc., USA) to obtain the pCa_50_ (where force reaches half-maximal force) and the Hill coefficient (*n*H), which represents the slopes of the curves. The pCa_50_ obtained before (5.73 ± 0.22) and after H_2_O_2_ treatment (5.70 ± 0.26) were not statistically different (p = 0.98). The *n*H of the fitted curves were also not different (p = 0.96) before (3.21 ± 0.36) and after (3.20 ± 0.55) treatment with H_2_O_2_.

## Discussion

The main findings of this study were that (i) isolated fibers from the mouse diaphragm developed a prolonged fatigue in response to low-frequency stimulation, that ultimately led to a decrease in force in all frequencies of stimulation ranging from 1 Hz to 150 Hz, (ii) treatment of H_2_O_2_ accentuated fatigue in all levels of stimulation, and (iii) H_2_O_2_ decreased the myofibril forces in different Ca^2+^ concentrations, without changing the Ca^2+^ sensitivity of the contractile apparatus. Altogether, these results suggest that fatigue in the diaphragm is caused partially by oxidation of the contractile proteins.

The pattern of force decline that we observed during the development of fatigue in this study was similar to that described in earlier studies that used non-respiratory skeletal muscles^18,38–40^. It shows a fast decline in force during the first 25 contractions and a slower decline in force during the subsequent 600 contractions. Although this finding might have been expected, it is relevant for the understanding of diaphragm fatigue, as studies with isolated, intact diaphragm fibers  have not been performed previously. Furthermore, there was a downward shift in the force-frequency relationship, showing that after fatigue the fibers needed higher levels of stimulation to produce similar levels of force as before fatigue; in fact the diaphragm fibers  produced ~60–80% of maximal force in frequencies ranging between 25 Hz and 75 Hz after fatigue (the range of physiological frequencies of activation), which is also similar to previous studies eliciting long-lasting fatigue in skeletal limb muscles^41^. Therefore, many characteristics of fatigue that have been documented in the literature using non-respiratory skeletal muscles fibers  seem to be valid for diaphragm fibers.

Treatment with H_2_O_2_ decreased the force in all frequencies investigated in this study, showing a strong effect of oxidation in the active force produced by the fibers. Comparisons with previous studies are challenging, as the direct effects of H_2_O_2_ on force production have been debated in the literature. In a study that investigated the mechanisms whereby ROS affects contractile function, intact mouse fast-twitch fibers were exposed to H_2_O_2_ (100–300 μM), which led to a transiently increased force production followed by a progressive force decrease, which could be reversed by the reducing agent dithiothreitol^42^. The result is similar to our observations with isolated myofibrils, where we saw a decrease in force after a transitory increase after administration of H_2_O_2_. Thus, under these experimental conditions, the contractile function became severely impaired in the oxidized state.

However, studies with skinned fibers show varying results, and in some cases H_2_O_2_ was observed to be relatively unreactive^43,44^. In one study, application of 10 μM H_2_O_2_ in muscle fibers from the rat limb for 5 min did not affect the maximum force or the Ca^2+^ sensitivity of the contractile apparatus - the only change observed in that study was a decrease in the steepness of the force-Ca^2+^ relationship^44^. However, when higher concentrations or longer exposure times to H_2_O_2_ were used (20 minutes instead of 5 minutes), the maximum force and Ca^2+^ sensitivity were decreased^44^. These data are in accordance with results obtained during a study showing that when the endogenous formation of H_2_O_2_ was facilitated by a high SOD_2_ activity, fatigue was caused by a decreased Ca^2+^ sensitivity of the contractile apparatus^32^. Therefore, it seems that both endogenously produced and exogenously applied H_2_O_2_ may affect myofibrillar function, depending on the experimental condition. These results are also in qualitative agreement with our results with fibers and myofibrils. We observed that treatment with H_2_O_2_ decreased the force in intact fibers almost instantaneously, while treatment with H_2_O_2_ first increased the force in isolated myofibrils (after 5 minutes), to then decrease it significantly (after 15 minutes). The authors^44^ also observed a decrease in force after 20 minutes of exposure to H_2_O_2_, which is consistent with our results, although direct comparisons are difficult due to differences in experimental conditions (temperature, pH, type of muscle samples). For example, the studies performed with skinned fibers from limb muscles observed different effects of H_2_O_2_ on fast and slow-twitch muscle fibers. The diaphragm has a complex fiber type distribution, with fast and slow fibers^45,46^, and the effects of H_2_O_2_ on force production and Ca^2+^ sensitivity may reflect such heterogeneity.

We observed that H_2_O_2_ combined with fatigue decreased the force levels significantly more, even after recovery, in all frequencies investigated; at 150 Hz force was still ~80 of maximal force produced before fatigue and H_2_O_2_ treatment. This result strongly suggests that oxidation, which happens normally during repeated muscle contractions, can play a major role in prolonged muscle fatigue. The mechanism underlying prolonged low-frequency fatigue depended on the capacity of muscle fibers to convert O_2_^−•^ to H_2_O_2_ via the mitochondrial superoxide dismutase (SOD_2_)^32^. Our results are in agreement with experiments with single fast-twitch fibers in which fatigue was caused by a decreased Ca^2+^ sensitivity in rat fibers with higher SOD_2_ capacity and in mouse fibers overexpressing SOD_2_ (i.e. promoting accumulation of H_2_O_2_), whereas it was caused by decreased SR Ca^2+^ release in mouse fibers with relatively low SOD_2_ capacity (i.e. promoting accumulation of O_2_^−•^).

The decrease in the myofibril force shows that oxidation affects directly the contractile system in the muscle fibers. The myofibril preparation eliminates all the steps in the excitation-contraction coupling and Ca^2+^ release from the sarcoplasmic reticulum, and therefore the decrease in force with H_2_O_2_ treatment is likely associated with modification in the myosin and/or actin proteins, without affecting the Ca^2+^ sensitivity of the contractile apparatus. It has been shown that oxidative modifications in muscle fibers has been associated with impaired myofibrils kinetics in some diseases and abnormalities, including inflammation and rheumatoid arthritis^47,48^ and ventilatory-induced diaphragm dysfunction (VIDD)^49,50^. It is tempting to suggest that any change in muscle activity that leads to oxidation, like fatigue, may decrease force production in skeletal muscles by directly affecting the molecular interactions between myosin and actin filaments.
